# Early prediction of hemodynamic interventions in the intensive care unit using machine learning

**DOI:** 10.1186/s13054-021-03808-x

**Published:** 2021-11-14

**Authors:** Asif Rahman, Yale Chang, Junzi Dong, Bryan Conroy, Annamalai Natarajan, Takahiro Kinoshita, Francesco Vicario, Joseph Frassica, Minnan Xu-Wilson

**Affiliations:** 1 Philips Research North America, Cambridge, MA 02141 USA; 2 Institute for Medical Engineering and Science, Massachusetts Institute of Technology, Cambridge, MA 02139 USA

**Keywords:** Hemodynamics, Vasoactive therapy, Machine learning, Clinical decision support

## Abstract

**Background:**

Timely recognition of hemodynamic instability in critically ill patients enables increased vigilance and early treatment opportunities. We develop the Hemodynamic Stability Index (HSI), which highlights situational awareness of possible hemodynamic instability occurring at the bedside and to prompt assessment for potential hemodynamic interventions.

**Methods:**

We used an ensemble of decision trees to obtain a real-time risk score that predicts the initiation of hemodynamic interventions an hour into the future. We developed the model using the eICU Research Institute (eRI) database, based on adult ICU admissions from 2012 to 2016. A total of 208,375 ICU stays met the inclusion criteria, with 32,896 patients (prevalence = 18%) experiencing at least one instability event where they received one of the interventions during their stay. Predictors included vital signs, laboratory measurements, and ventilation settings.

**Results:**

HSI showed significantly better performance compared to single parameters like systolic blood pressure and shock index (heart rate/systolic blood pressure) and showed good generalization across patient subgroups. HSI AUC was 0.82 and predicted 52% of all hemodynamic interventions with a lead time of 1-h with a specificity of 92%. In addition to predicting future hemodynamic interventions, our model provides confidence intervals and a ranked list of clinical features that contribute to each prediction. Importantly, HSI can use a sparse set of physiologic variables and abstains from making a prediction when the confidence is below an acceptable threshold.

**Conclusions:**

The HSI algorithm provides a single score that summarizes hemodynamic status in real time using multiple physiologic parameters in patient monitors and electronic medical records (EMR). Importantly, HSI is designed for real-world deployment, demonstrating generalizability, strong performance under different data availability conditions, and providing model explanation in the form of feature importance and prediction confidence.

**Supplementary Information:**

The online version contains supplementary material available at 10.1186/s13054-021-03808-x.

## Introduction

Fluid resuscitation and vasoactive therapy are essential in the management of hypotensive patients to support organ perfusion [1–3]. Current guidelines from the 2016 Surviving Sepsis Campaign (SSC) recommend early initiation of vasopressors targeting a mean arterial pressure ≥ 65 mmHg [4]. According to the guidelines, the need for initiation of vasopressor therapy should be assessed if there is ongoing hemodynamic instability despite fluid resuscitation. Although the SSC guidelines are not precise about the appropriate time to initiate vasopressors, recent studies have demonstrated that delayed initiation of vasopressors is associated with higher mortality, fewer vasopressor-free days, and longer time to achieve target mean arterial pressure [5, 6].

Clinical decision support systems that are designed to continuously monitor and identify patients at a high risk of developing hemodynamic instability have the potential to improve the timely recognition of the need for immediate pressure support [7, 8]. Early initiation of hemodynamic interventions based on these systems can potentially help avoid complications from organ hypoperfusion and reduce mortality. Commonly used single parameter measurements including blood pressure and heart rate are easily acquired at the bedside and can be used as a risk stratification tool for detecting changes in hemodynamic parameters [9]. However, single parameter monitoring does not fully describe the entire patient state and can potentially lead to misinterpretation and underestimation of instability. Multi-parameter scoring systems using machine learning to quantify associations between physiologic variables and adverse events have been proposed as a way to accurately stratify ICU patients.

Hemodynamic interventions including the initiation of vasopressors or inotropes, fluid administration, and blood transfusions are markers of significant hemodynamic instability in ICU patients. In this study, we aimed to (1) develop a multiparameter risk score that stratifies patients with a high probability of receiving a hemodynamic intervention. (2) Identify the important physiological parameters that contribute to the risk and quantify the confidence of the model predictions. (3) Evaluate model performance on subgroups of ICU patients and on an independent validation cohort.

## Methods

**Fig. 1:**
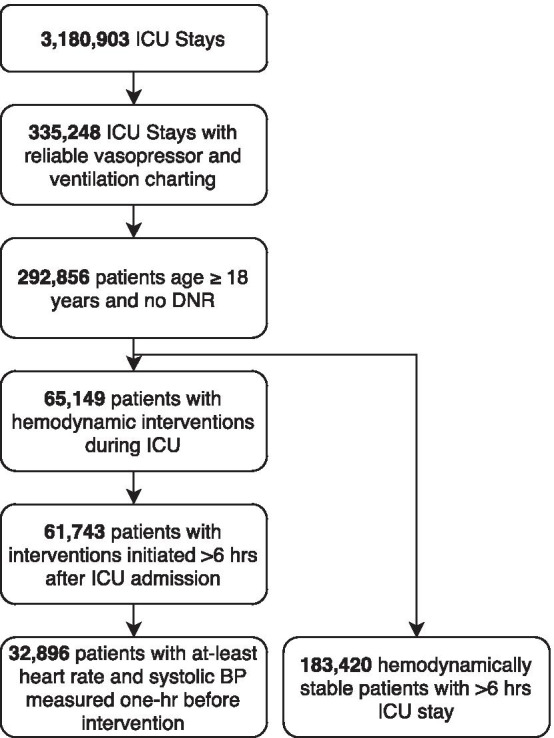
Extraction of HSI cohort

We developed a machine learning model using retrospective data from patients in the ICU to predict the onset of hemodynamic interventions one-hour in the future. The eICU Research Institute (eRI) database was used for the purposes of training and validation (Pollard et al.). The full dataset is comprised of 3.3 million patient encounters from 364 ICUs across the USA. To ensure that charting of hemodynamic intervention data was accurate in the training and validation cohorts, we restricted our analysis to patients admitted to hospitals with reliable infusion and ventilation charting data between 2012 and 2016. Hospitals were considered reliable if they had charted ≥ 7 infusion drug entries per patient per day, included patients with ≥ 0.75 ventilation and airway records per patient per day in the patient care plan, and ≥ 10 entries per patient per day in respiratory charting tables in eRI database. We further limited our cohort to adult patients ≥ 18 years old who did not have a do not resuscitate (DNR) indication in the ICU. This filtering step reduced the initial dataset size to 292,856 patient encounters from 54 ICUs (Fig. 1).

ICU patients were classified into stable and unstable groups. Stable patients did not receive any of the hemodynamic interventions in Table 1. Unstable patients received at least one of the interventions in Table 1 during the ICU stay, including the initiation of pressors or inotropes, administration of a significant dose of fluids in a short time period, or packed red blood cell (PRBC) transfusions. An intervention segment started when any of the intervention criteria was satisfied [10–12]. The intervention segment continued until there was a gap of more than 12 h between consecutive pressors or inotropes, fluid administrations, or PRBC transfusions. The last set of physiological variables observed 1-h before intervention was used as a positive class sample and a random time from a stable patient was selected as the negative class sample for model training. We did not include any samples from the first 6 h of the ICU stay in either the hemodynamically stable or unstable groups during training. A stratified subsample of 20% of the eRI data were held out and reserved for model evaluation, while the remaining 80% were used to train the model. Samples were stratified so that a patient appears in only one of the train or test sets, but not both. Additionally, we validated the model trained on eRI patients on an external dataset from an independent hospital, namely the MIMIC III database [13]. We extracted the stable and unstable samples from MIMIC III following the same process described above, however, the outcome label included only pressor or inotrope administration.

**Table 1:** Criteria used to define hemodynamic instability

A segment was labeled “intervention” under any of the following conditions	
Administration of any quantity of any of the following inotropic and vasopressor medications: Dobutamine Dopamine Epinephrine Norepinephrine Phenylephrine Vasopressin Administration of Fluid Therapy (colloid or crystalloid) in the following dosages: 2400 cc in 8 h 3000 cc in 12 h 700 cc in 1 h 1500 cc total in 4 h 500 cc twice in 4 h Administration of Packed Red Blood Cells (PRBCs) in either of the following dosages: 800 cc PRBC over course of 24 h 500 cc in two hours followed by fluid therapy within 12 h. (What qualifies as “fluid therapy” is described in this table, titled “Administration of Fluid Therapy.”) 500 cc PRBC not followed by fluid therapy within the following 24 h. (What qualifies as “fluid therapy” is described in this table entry titled “Administration of Fluid Therapy.”)	

The fluid trigger criteria were derived from clinical consensus of a panel of clinical experts in fluid and hemodynamic management. Some are multiples of standard dosing regimens (10 cc/kg, 20 cc/kg) or multiples of the size of bags of solution that are used for fluid resuscitation (500 cc or 1 L). The starting bolus for an adult is 500 cc OR 10 cc/kg. For significant hypovolemia, this might be 1400 cc (20 cc/kg) or 1 L (the size of a 1-L bag of solution). The fluid triggers represent what was considered a significant intervention in response to hypovolemia. Additional details describing the rationale for each fluid trigger can be found in the Additional file 1

### Clinical observations

We selected 33 variables that are routinely acquired in the ICU, including vital signs, laboratory measurements, blood gas measurements, and ventilation settings (Additional file 1: Figure S3). Variables were forward filled up to 2 h for heart rate and systolic blood pressure, and up to 26 h for laboratory measurements and ventilator settings. Invasive and noninvasive blood pressures were combined into a single variable with invasive blood pressure prioritized over noninvasive measurements when both are available. We require at least a heart rate and systolic blood pressure be available for the calculation of a risk score during training and evaluation. If a variable was missing because it was not measured or the forward filled value expired, the value was imputed using the training data population mean of ICU patients for all features except the three ventilation parameters: fraction of inspired oxygen (FiO2), mean airway pressure (MAWP), and positive inspiratory pressure (PIP). FiO2 is imputed to room oxygen level of 0.21, and MAWP and PIP are left as missing to avoid imputing ventilation settings for patients who were not mechanically ventilated.

### Supervised learning of hemodynamic interventions

We trained an Abstain-Boost model [11], which is a powerful ensemble of univariate classifiers composed of decision trees of depth one that predict future hemodynamic status (stable or unstable) based on individual patient measurements. Each of the 33 classifiers (one for each physiologic variable) outputs a real value, with more positive values indicating a greater risk for hemodynamic interventions. Variable-wise risks are summed and sigmoid transformed for the final probability of hemodynamic intervention. The model was trained with 200 rounds of boosting with learning rate set to 0.1. The predicted probabilities are calibrated using Platt scaling [14] after model training to match the empirical instability rate observed in the data. We define the hemodynamic stability index such that higher probability indicates a lower risk of hemodynamic interventions (stability). We include the TRIPOD Checklist to report model development and validation steps.

**Fig. 2:**
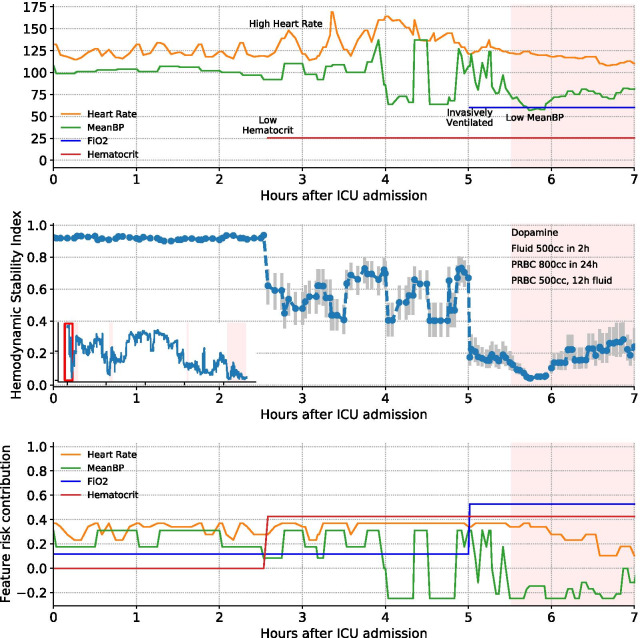
Illustrative patient case showing individual features (top) the hemodynamic interventions administered for this patient, the HSI model predictions with confidence intervals (middle), and univariate risk scores contributed by select features from the HSI model (bottom). There is an emergent hemodynamic situation within the first day of ICU admission leading to a blood transfusion along with fluid and dopamine administration. HSI acts as an early indicator by responding to a sudden decrease in blood pressure and initiation of invasive mechanical ventilation

We also calculate confidence intervals to quantify uncertainty in model predictions. Figure 2 shows the HSI score along with confidence intervals for an illustrative patient case. Uncertainty in model predictions can be decomposed as model uncertainty, which is the level of uncertainty derived from model under-specification (e.g., if the algorithm does not capture nonlinear relationships), and from feature uncertainty, which is driven by noisy measurements and missing variables. We quantify these sources of uncertainty to calculate confidence intervals (see Additional file 1 for details). The model can abstain from making predictions based on the degree of overlap between the confidence interval and a critical threshold of the HSI risk score where patients transition from stable to unstable. A high degree of overlap between the confidence interval and the critical threshold indicates greater uncertainty about whether the patient needs an intervention and thus we can abstain from making a prediction. See Additional file 1 for technical details and experiments on abstention.

Clinical risk prediction models are susceptible to learning patterns of clinical actions and not just the patient physiology [15]. During model training, we attempted to remove the bias from clinicians’ actions by (1) merging invasive and noninvasive blood pressure to remove the influence of the invasive measurement. The presence of invasive measurements indicates higher clinical concern, and the model would learn to assign higher risk simply based on the presence of the invasive variable. (2) Missing values were mean imputed with the population mean so the model is not learning from missingness patterns. (3) We experimented with adding missing variable indicators to the model and it improved model performance. However, we decided to exclude missing-variable indicators to learn purely from the physiology and not patterns of clinical practice.

### Evaluation

We report model performance using the area under the receiver operator curve (AUC); sensitivity (Se) also known as recall, which is the model’s capacity at predicting the hemodynamic interventions received by patients; specificity (Sp), which quantifies the false predictions of a hemodynamic intervention when the patient did not receive one; and the positive predictive value (PPV) also known as precision, which is the fraction of all predictions that truly resulted in an intervention. Performance metrics are reported at the breakeven point (BE), where precision equals recall, at 90%, and at 95% specificity. The model, trained using all 33 input variables, was evaluated under four distinct operating modes representative of realistic hospital deployment conditions with varying levels of integration of different data sources: (1) a “Basic” mode where the model has access to a small set of vital signs including heart rate, blood pressures, shock index and age, (2) a “Basic + Labs” mode where available laboratory measurements are used by the model in addition to variables from the basic mode, (3) a “Basic + Ventilation” mode where ventilator settings, when available, are used in addition to basic mode variables, and (4) an “All Features” mode where all available variables are presented to the predictive model. Operating modes were simulated by treating variables that are not included in the respective operating modes as missing values. We also report model performance on patient subgroups, including ICU stay type (e.g., stepdown, transfer from general ward, readmission), ICU unit type (e.g., Med-Surg, Cardiac), admission source (e.g., Floor, ICU), and ventilation status at the time of prediction.

## Results

The cohort selection criteria (Fig. 1) identified 32,896 unstable events leading to a hemodynamic intervention and 183,420 stable events where patients did not receive any hemodynamic intervention (prevalence = 18%). Patients in the unstable group had longer ICU length of stay (median; Stable: 29 h; Unstable: 95 h), more days on invasive mechanical ventilation (Stable: 22 h; Unstable: 75 h), greater hospital mortality rate (Stable: 1.9%, Unstable: 9.0%), and had higher APACHE IV score at ICU admission (Stable: 46, Unstable: 62). Of the 32,896 unstable events, 19,044 resulted in pressor administration (58%), 5,159 events resulted in PRBC transfusions (16%), and 11,918 events resulted in significant fluids (36%).

**Fig. 3:**
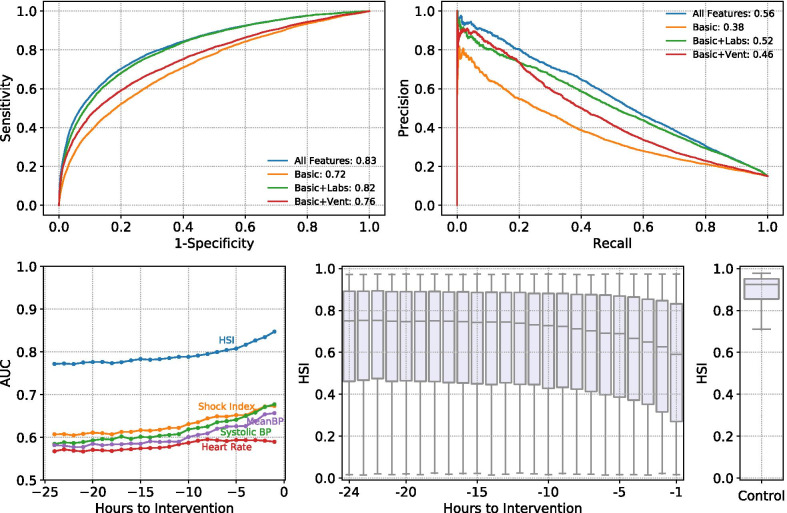
HSI model performance at different operating modes and at different prediction times before hemodynamic interventions. Legends on the receiver-operator curve (ROC) and the precision-recall curve (PRC) report the AUC and AUPRC, respectively. Laboratory measurements gave a significant increase in model performance (10% increase in AUC and 14% increase in AUPRC) compared to using basic vital signs like heart rate, blood pressures, and shock index

Using the available measurements from all 33 physiologic variables, the HSI model has an AUC of 0.82 (Sp = 0.92, PPV = 0.52 at the breakeven point) on the held-out dataset from the eRI database when predicting all outcomes including pressors or inotropes, fluids, and PRBC administrations (prevalence = 15%) one hour before the event. The AUC improves to 0.88 (Sp = 0.95, PPV = 0.55) in predicting pressor administrations alone (prevalence = 11%) (Table 2). HSI has high predictive accuracy even up to 12 h prior to the event and significantly outperforms single parameters like shock index and systolic blood pressure in predicting hemodynamic interventions (Fig. 3).

**Table 2:** HSI model performance (all features operating mode)

Outcome	AUC	Sp (BE)	PPV (BE)	Se (Sp = 90%)	PPV (Sp = 90%)	Se (Sp = 95%)	PPV (Sp = 95%)
Pressors, fluids, PRBC	0.82	0.92	0.52	0.55	0.49	0.43	0.6
Pressors (11%)	0.88	0.95	0.55	0.68	0.44	0.55	0.56

### Model performance with missing variables

HSI was able to predict instability accurately under more restricted data conditions where some measurements were not available, as shown in in Table 3 (see “Evaluation” section in “Methods” for a detailed definition of operating modes). The AUC decreases to 0.72 (PPV: 0.39, AUPRC) when only age, heart rate, blood pressures, and shock index (Basic mode) are available and laboratory measurements and ventilation settings are treated as missing variables, still outperforming blood pressure and shock index. Laboratory measurements are responsible for an 8% increase in AUC when we compare the Basic mode to the Basic + Labs mode (AUC from 0.72 to 0.8; PPV from 0.39 to 0.48).

**Table 3:** HSI trained on all features is evaluated under specific operating modes where limited set of features are accessible to the model

Operating mode	AUC	Sp (BE)	PPV (BE)	Se (Sp = 90%)	PPV (Sp = 90%)	Se (Sp = 95%)	PPV (Sp = 95%)
All features	0.82	0.92	0.52	0.55	0.49	0.43	0.6
Basic	0.72	0.89	0.39	0.37	0.4	0.26	0.48
Basic + labs	0.8	0.91	0.48	0.5	0.47	0.37	0.57
Basic + ventilation	0.76	0.9	0.45	0.46	0.45	0.35	0.55

The outcome label includes pressors, fluid, and PRBC administration. We report the area under the receiver operator curve (AUC), Specificity (Sp), Sensitivity (Se) and Positive Predictive Value (PPV) at the breakeven point (BE) where precision equals recall, and at both 90% and 95% Specificity

### Model performance in patient subgroups

We verified that the HSI model generalizes well across different patient groups defined by ICU stay type, ICU unit type, admission source, and ventilation status. As reported in Additional file 1: Table S1, HSI performs significantly worse in stepdown units (PPV decreases from 0.529 to 0.146) where there was low prevalence of hemodynamic interventions and in neurological ICUs. Detailed analysis is given in Additional file 1.

### External validation

HSI was externally validated on MIMIC III database, which is independent from the eRI database used for training. We identified 15,981 ICU stays matching our extraction criteria following the same procedure as in the eRI database. The outcome label included patients that received pressors or inotropes but not fluids and PRBC. The prevalence was significantly higher in the MIMIC III database (37.8%). We observed a higher AUC 0.90 in MIMIC III (PPV: 0.79, Sp: 0.87 at the breakeven point) when evaluated one-hour prior to intervention. Adjusting the MIMIC III prevalence to the eRI prevalence of 11% of pressors—only by subsampling—gave an AUC of 0.90 (PPV: 0.61, Sp: 0.95). These results suggest that our model trained from various hospitals is generalizable to different institutions and care settings.

### Feature importance

**Fig. 4:**
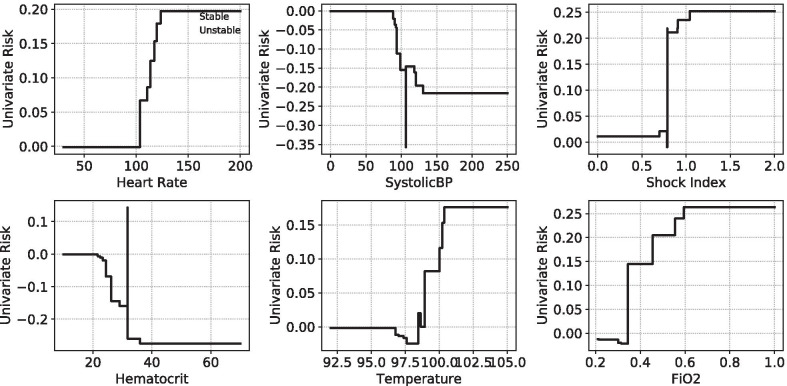
Examples of univariate risk curves (black) and feature distribution histograms for stable (blue) and unstable (orange) patients

Global feature importance can be visualized as risk curves like in Fig. 4. HSI learns that early physiological signs of shock, including elevated heart rate and low blood pressure, increase the risk of a hemodynamic intervention. Lower than normal hematocrit levels, indicating an insufficient supply of healthy red blood cells, leads to a higher risk of hemodynamic interventions like blood transfusions. Figure 2 shows for an example patient the univariate risk scores for individual physiologic variables. Univariate risks (which are added to calculate the total HSI score) are used to identify the top features contributing to the risk and give caregivers context for the prediction as well as cues for how to react to it.

## Discussion

The HSI model provides an early warning of hemodynamic instability by detecting the need for significant hemodynamic interventions. The major finding of the current study was that HSI, a novel, multi-parameter machine learning model, far outweighed traditional metrics such as shock index and systolic blood pressure at predicting the need for hemodynamic interventions. Although the discrimination accuracy was best when used to predict hemodynamic interventions 1-h before the events, it was also highly predictive even at 12-h before the initiation of the hemodynamic interventions. Importantly, HSI learns clinically meaningful and interpretable relationships between physiological variables and the risk of a hemodynamic intervention.

HSI generalizes well across most subgroups and in an independent validation cohort. On the external validation dataset where the outcome label included only pressors, HSI had the same AUC as our held-out evaluation data in the eRI database. However, the performance on patients in the stepdown unit was worse than other units. This is because there exists significant mismatch between feature distributions and label distributions of the stepdown units and those of the general ICU patient population. Specifically, we find the unstable patient group that received a hemodynamic intervention in the stepdown units are physiologically more stable with higher systolic blood pressure and lower prevalence of ventilation. These factors lead to a lower predicted risk of hemodynamic interventions in the unstable patients of the stepdown units (higher rate of false negatives), making the separation of the unstable patients from stable patients using HSI more difficult on stepdown patients.

Similarly, although HSI has strong predictive performance on most ICU units, it had a low AUC and PPV on neurological ICU patients. In the unstable group, neurological ICU patients have significantly lower risk of hemodynamic instability than patients in other care units, and as a result, the model has a lower true positive rate. Unstable patients in neurological ICUs have significantly higher systolic blood pressure, lower heart rate, higher hematocrit, and hemoglobin. Clinically, neurological patients are intentionally made hypertensive using vasopressors, which overlap with those used to define interventions for the model. The administration of vasopressors to neurological ICU patients does not necessarily indicate the onset of hemodynamic instability but reflects routine treatment patterns in patients admitted to the neurological ICU unit.

Our work shares some similarities with prior work on early detection of adverse hemodynamic events where pressor administration was used as an outcome label as a surrogate marker of hemodynamic instability [8, 16–18]. In contrast to prior work, we defined hemodynamic interventions using a broader category of treatments including significant fluid administration within a short time and blood transfusions with PRBC in addition to pressor or inotrope initiation. Hyland et al. (2020), for example, defines circulatory failure using thresholds on lactate, mean arterial pressure, and administration of vasopressors or inotropes. Our definition of an adverse hemodynamic event captures a more general case. For instance, our labels include the case where resuscitation leads to a rapid increase in fluid administration in a short period of time before pressors are initiated. Other technical differentiators between HSI and prior work are that we achieve good predictive performance and generalization using a few commonly measured vital signs and laboratory measurements. In contrast to prior work, our model also provides confidence intervals, can abstain from making predictions when uncertainty is high, and is inherently interpretable because we use an ensemble of decision stumps. This contrasts with Hyland et al. (2020) where the final model uses an ensemble of deep decision trees with 4-levels of interactions and relies on post hoc explanation methods (Shapley values) to provide a global feature importance. The TREWScore is another alternative to HSI, designed to predict the onset of septic shock [19]. The TREWScore was developed on a cohort or sepsis patients, unlike HSI which is trained on a larger more heterogenous patient population, including patients with septic shock. We hypothesize that the adjunct analyses (operating modes, subgroups) and algorithm enhancements (confidence intervals, abstention, feature importance) we described will support deployment of HSI and similar decision support algorithms in real clinical settings.

HSI has been trained by learning from clinician’s actions such as administration of vasopressors, inotropes, fluids, and PRBCs. The approach follows the rationale that clinicians’ decisions to intervene consider broad and diverse information about the patient (part of which is not even captured or not captured timely in EMR systems), of which the experienced clinician makes sense of, due to their years of training and experience. By learning from clinicians’ actions on thousands of patients rather than from arbitrary definitions of hemodynamic instability events based on physiological or laboratory measurements crossing a fixed, pre-defined one-size-fits-all threshold, HSI gets one step closer to personalized care. Additionally, HSI uses the result of a laboratory test instead of the presence (or absence) of a laboratory test to model patient physiology instead of the institution-specific care pattern [15].

The present study has several limitations. First, our model is tested on retrospectively collected datasets only. However, using a training dataset that captures practice variations of ICUs all over the U.S. gives the algorithm a good chance of being generalizable. Furthermore, we show high external validity of the predictive performance on an external dataset and on patient subgroups, suggesting potential generalizability. Second, an advantage of HSI—using a limited set of physiologic variables—can also be considered a limitation because we lack advanced hemodynamic measurements like cardiac output, stroke volume and stroke volume variation, which would likely add predictive power to HSI and make HSI more applicable in assessing fluid responsiveness [7, 20]. We also don't include medication information in our model. Intuitively, certain physiologic parameters could be conditionally dependent on medications. Future work will focus on prospective validation of HSI in the ICU setting to show that such a system can impact patient outcome.

## Conclusions

We developed an accurate and automated early prediction algorithm to identify ICU patients at risk of developing hemodynamic instability using commonly measured physiological variables. The HSI model demonstrates generalizability across ICU units, patient subpopulations, institutions, and operating modes. Importantly, we develop the algorithm into a decision support tool that provides interpretable feature importance, measures uncertainty in real time, abstains from making predictions with high uncertainty, and gives actionable prompts to take new measurements based on a feature impact score. The analysis and supporting algorithms presented around HSI will be especially critical in real-world deployment scenarios that require good generalizability, handing of different data availability, and explanation of algorithm output in the form of feature importance and prediction confidence.

## Supplementary Information

**Additional file 1.** Supplementary Methods.

## Data Availability

The datasets generated and/or analyzed during the current study are available in the eICU and MIMIC repositories, https://eicu-crd.mit.edu/, https://mimic.physionet.org/.

## Abbreviations

eRI: EICU Research Institute
ICU: Intensive care unit
HSI: Hemodynamic stability index
FiO2: Fraction of inspired oxygen
AUC: Area under the curve
Sp: Specificity
Se: Sensitivity
PPV: Positive predictive value
BE: Breakeven
