# Supplementary material for: Early prediction of hemodynamic interventions in the intensive care unit using machine learning
Source: Crit Care. 2021 Nov 14;25:388. doi: 10.1186/s13054-021-03808-x (PMC8590869; doi:10.1186/s13054-021-03808-x)
Supplement: Supplementary file 1 — Additional file 1. Supplementary Methods. [file 13054_2021_3808_MOESM1_ESM.docx]

**SUPPLEMENTARY MATERIALS: Early prediction of hemodynamic interventions in the intensive care unit using machine learning**

***Subgroup performance***

HSI generalizes to different unit stay types including direct admission, readmissions, and transfers. However, HSI performs significantly worse in stepdown units where the breakeven precision-recall decreases from 0.529 to 0.146. It is to be noted that the prevalence of unstable stepdown patients is extremely low in the dataset used to train HSI (14% of the total patients are stepdown and only 2% of these experience hemodynamic instability). The performance of HSI is high across unit types, with the exception of neurological ICU (Supplementary Table 1). This finding is consistent with the practice of deliberate hypertension in neurosurgical patients, recommended by clinical guidelines to protect the brain from potential hypoperfusion. Patients admitted from the emergency department, floor, and operating room have the highest prevalence in our dataset and HSI has good predictive power in this cohort. Patients admitted from chest pain center, from another ICU, from ICU to stepdown unit, from post-anesthesia care unit, or from a recovery room have low prevalence in our dataset and performance varies across these subgroups as see in Supplementary Table 1. Specifically, patients from chest pain center and post-anesthesia care units have extremely low prevalence of 0.2% and 1.6%, respectively. In the unstable group, patients admitted from ICU to stepdown units and recovery room have significantly higher systolic blood pressure and are less likely to be invasively ventilated, two features that consistently rank high as contributors to the HSI predictions. HSI performance also varies by ventilation status. HSI does better at detecting interventions in invasively ventilated patients than in patients that are not ventilated at the time of prediction (Supplementary Table 1).

Table 1: HSI evaluated on subgroups of patients. AUC=area under receiver operator curve, AUPRC=area under precision recall curve, PPV=positive predictive value.

| **Subpopulation** | **Percentage of patients** | **Prevalence of unstable patients** | **AUC** | **AUPRC** | **PPV** |
| --- | --- | --- | --- | --- | --- |
| **ICU Stay Type** | | | | | |
| All | 100% | 15% | 0.827 | 0.556 | 0.529 |
| Direct Admit | 71% | 16% | 0.817 | 0.567 | 0.534 |
| Readmit | 5% | 22% | 0.822 | 0.627 | 0.595 |
| Stepdown | 14% | 2% | 0.753 | 0.1 | 0.146 |
| Transfer | 7% | 19% | 0.811 | 0.59 | 0.552 |
| **ICU Unit Type** | | | | | |
| All | 100% | 15% | 0.827 | 0.556 | 0.529 |
| Coronary care unit – cardiothoracic ICU | 5% | 17% | 0.824 | 0.582 | 0.551 |
| Cardiac surgery ICU | 11% | 11% | 0.859 | 0.558 | 0.536 |
| Cardiothoracic ICU | 0% | 28% | 0.803 | 0.703 | 0.657 |
| Cardiac ICU | 6% | 7% | 0.889 | 0.555 | 0.551 |
| Medical ICU | 8% | 23% | 0.785 | 0.599 | 0.534 |
| Medical-Surgical ICU | 54% | 13% | 0.827 | 0.54 | 0.52 |
| Neurological ICU | 2% | 18% | 0.706 | 0.441 | 0.408 |
| Surgical ICU | 6% | 23% | 0.839 | 0.658 | 0.598 |
| Trauma ICU | 2% | 15% | 0.847 | 0.562 | 0.52 |
| **Admission Source** | | | | | |
| All | 100% | 15% | 0.827 | 0.556 | 0.529 |
| Acute Care/Floor | 3% | 18% | 0.85 | 0.648 | 0.585 |
| Chest Pain Center | <1% | 10% | 0.752 | 0.303 | 0.3 |
| Direct Admit | 5% | 19% | 0.846 | 0.632 | 0.574 |
| Emergency Department | 42% | 13% | 0.819 | 0.537 | 0.515 |
| Floor | 10% | 20% | 0.809 | 0.59 | 0.548 |
| ICU | 4% | 4% | 0.891 | 0.419 | 0.452 |
| ICU to stepdown unit | 7% | 2% | 0.733 | 0.1 | 0.148 |
| Observation | <1% | 37% | 0.6 | 0.532 | 0.667 |
| Operating Room | 10% | 22% | 0.799 | 0.602 | 0.551 |
| Post-anesthesia Care Unit | 1% | 8% | 0.852 | 0.433 | 0.419 |
| Recovery Room | 4% | 13% | 0.71 | 0.377 | 0.378 |
| Stepdown Unit | 3% | 20% | 0.821 | 0.591 | 0.593 |
| **Ventilation Status** | | | | | |
| All | 100% | 15% | 0.827 | 0.556 | 0.529 |
| Off ventilation | 84% | 9% | 0.78 | 0.361 | 0.391 |
| On ventilation | 15% | 42% | 0.805 | 0.761 | 0.692 |

***Confidence intervals***

In binary classification with class label {stable (-1), unstable (+1)}, we use $r\left( x \right)=p(y=1|x)$ to represent the probability of the input sample $x\in\mathbb{R}^{D}$ is predicted to be in the unstable class. $r(x)$ is often created by applying the sigmoid function to its logit (log-odds) score $f(x)$, which is defined as follows

$$f\left( x \right)=\mathrm{logit}\left( r\left( x \right) \right)=\log\frac{r(x)}{1-r(x)}$$

We are interested in learning the predictive distribution of $f(x)$. Suppose the predictive distribution of $f(x)$ can be approximated by a Gaussian distribution $f(x)\sim\mathcal{N(}\mu\left( x \right),\sigma^{2}(x))$. The 95% confidence interval of HSI score, which is defined as $1-\mathrm{sigmoid}(f(x))$, can be written as

$$\mathrm{CI}_{95\%}\left( \mathrm{HSI}\left( x \right) \right)=[1-\mathrm{sigmoid}\left( \mu\left( x \right)+2\sigma\left( x \right) \right), 1-\mathrm{sigmoid}\left( \mu\left( x \right)-2\sigma\left( x \right) \right)]$$

We approximate the distribution of $f(x)$ by training $M$ (we set $M=1000$ in practice) models $\{f_{1}\left( x \right),\cdots f_{M}(x)\}$ using $M$ bootstrapped datasets (Supplementary Figure 1). However, it would be too costly to deploy these $M$ bootstrapped models to patient monitors because both the computational and storage cost would grow linearly with respect to $M$. To tackle this challenge, we applied knowledge distillation (1) to train two regressors, denoted as $h_{\mu}(x)$ and $h_{\sigma}(x)$ to approximate the mean and standard deviation of $M$ logit scores output by $M$ models. In this way, only two regressors need to be deployed, resulting in significantly reduction of computational and storage cost ($2/M$ of the cost of deploying $M$ models). The R-squared score of the mean regressor and the standard deviation regressor are 0.989 and 0.859 respectively. After training the distillation model, the approximated 95% confidence interval of HSI score can be computed as following

$$\mathrm{CI}_{95\%}\left( \mathrm{HSI}\left( x \right) \right)=[1-\mathrm{sigmoid}\left( h_{\mu}\left( x \right)+2h_{\sigma}\left( x \right) \right), 1-\mathrm{sigmoid}\left( h_{\mu}\left( x \right)-2h_{\sigma}\left( x \right) \right)]$$

We compute the probability that $\mathrm{CI}_{95\%}\left( \mathrm{HSI}\left( x \right) \right)$ covers the prediction of the trained abstain adaboost model (after applying probability calibration) as 0.944. Therefore, it is practical to display the confidence interval around the decision function output by a single abstain-adaboost model with calibration. The model can abstain from making predictions if the 95% confidence interval covers the cutoff, implying the HSI score could either reach above or fall below the cutoff. We would demonstrate this strategy could effectively improve the classification performance in the experimental results.

***Confidence Interval and Feature Impact Score***

The HSI model triggers a notification by applying a fixed threshold (breakeven point where precision equals recall) to the HSI score. However, the model could make overconfident predictions for 1) patients closer to the decision boundary; and 2) where important features are missing (Supplementary Figure 2). In both cases, it would be desirable for the model to output high prediction uncertainty around the risk score, implying it’s highly probable that the model’s prediction is incorrect.

Figure 1. Predicted variance is decomposed into model and input uncertainty.

Figure 2. Input and model uncertainty can be used to flag predictions that have high confidence. Predictions that have poor input quality can trigger an alert to get the latest measurements for the variables with high input uncertainty.

To compute the confidence interval of HSI, we train 1000 different HSI models using bootstrapped datasets (Supplementary Figure 1). The mean and standard deviation of multiple predictions of these bootstrapped models can be used to construct the 95% confidence interval of HSI score. Furthermore, to reduce the memory and computational cost of running 1000 models during the test time, we apply knowledge distillation to learn two regressors to approximate the mean and standard deviation of multiple predictions. For a given threshold, the classifier can abstain from making predictions for samples whose confidence interval covers the threshold. In practice, we prefer setting an upper bound on the abstention rate, denoted as maxAbstentionRate: among all patients whose confidence intervals cover the threshold, the classifier can choose to abstain from making predictions for patients who have top-ranking distance between the lower/upper bound of the confidence interval and the threshold. We vary the budget of abstention from 0.05 to 0.2 and test how the abstention affects the precision/recall of the unstable class (Supplementary Figure 3). For example, setting the abstention budget to be 0.1, when the recall is equal to 0.61, abstention through confidence interval can increase the precision from 0.48 to 0.57.

Figure 3: the precision/recall curve of the HSI model without abstention (in blue) and with different values of the maximal abstention rate (in red, green, and magenta).

Since HSI wouldn’t be reliable if important features, such as blood pressure, are missing, we develop feature impact score (FIS) to quantify the patient-level feature importance of each missing feature. FIS is the reduction of input uncertainty, which is defined as the prediction variance due to the uncertainty of input data, if a missing feature is known. Clinicians are suggested to actively take measurements of those missing features having high FIS values, which can effectively improve the prediction performance due to the availability of important input features.

***Definition of Model Uncertainty***

The model uncertainty and input uncertainty are two sources contributing to the variance of $f(x)$. Models trained using patient cohorts collected from different hospitals would be different. Models trained using different subsets of the same hospital would also be different. During training, although we have access to patients collected from multiple hospitals, we still need consider the potential model variation using different patient cohorts, which can be simulated through bootstrapping. We define model uncertainty as the prediction variance due to the variation of the trained model $f_{\theta}(\cdot)$ given potential different training dataset, where $\theta$ represents the model parameters. The distribution of model parameters can capture the model variation induced by the variation of training datasets. Model uncertainty is useful because it would be higher for patients’ data closer to the decision boundary.

***Definition of Input Uncertainty***

We define input uncertainty as the prediction variance due to the imperfect quality of the input data, including missing features, unreliable feature values due to their old ages, and measurement noise. Denote the observed feature vector as $x$ and the processed feature vector as $z$, which is derived by applying pre-processing steps on $x$, including 1) imputation to fill in missing values, which can be used to quantify the influence of missing values on input uncertainty; and 2) renewing old-aged temporal measurements, which can be used to quantify the bias of old-aged measurements on input uncertainty. These pre-processing steps can be captured by $p(z|x)$, the conditional distribution of processed input $z$ given raw input $x$.

Input uncertainty is useful because it would be higher for patients whose input data quality is low. For example, if hemoglobin is missing for a patient the resulting high input uncertainty can be used to make the classifier abstain from making predictions for hemoglobin.

***Definition of Feature Impact Score***

We can further quantify the contribution of each feature to the input uncertainty by computing the feature impact score (FIS) for each feature, which is defined as the reduction of input uncertainty if the feature's quality is ideal. The FIS is useful because its values would be high for features that 1) are predictive of the outcome variable; and 2) have low quality (such as missingness). Therefore, we can rank features by their FIS values and suggest clinicians to improve the quality of features having high FIS values. Because these features are predictive of the outcome variable, improving their qualities (such as taking new measurements) would also improve the classification performance.

For example, consider the patient with a few lab values missing, the FIS of the missing lab values can be computed by the reduction of input uncertainty if these lab values were measured, which can be simulated through multiple imputation (Rubin 1988). In the missing feature example, we show FIS can be interpreted as patient-level feature importance score for missing features. After computing the FIS of these missing features, we suggest clinicians to measure variables having the highest FIS values. In this way, 1) the input uncertainty can be actively reduced; and 2) the classification performance can be improved.

***Decomposition of Prediction Variance***

The prediction variance can be decomposed as the summation of model uncertainty and input uncertainty (Rubin 1988). The decomposition can 1) provide formal definitions of these two kinds of uncertainties; and 2) illustrate how to estimate them given observed data.

The prediction mean $\mu(x)$ can be evaluated as

$$\mu\left( x \right)=\mathbb{E}_{p(z|x)}\mathbb{E}_{p(\theta)}[f_{\theta}(z)]$$

The prediction variance $\sigma^{2}(x)$ can be decomposed into model uncertainty and input uncertainty

$$\sigma^{2}\left( x \right)=\sigma_{model}^{2}+\sigma_{input}^{2}$$

where the model uncertainty can be evaluated as

$$\sigma_{model}^{2}=\mathbb{E}_{p(z|x)}[\mathrm{Var}_{p\left( \theta\right)}[f_{\theta}(z)]]$$

The input uncertainty can be evaluated as

$$\sigma_{input}^{2}=\mathrm{Var}_{p(z|x)}[\mathbb{E}_{p\left( \theta\right)}[f_{\theta}(z)]]$$

***Confidence Interval Based on Model Uncertainty***

$p(\theta)$, the distribution over model parameters, can be simulated by training $M$ models using $M$ bootstrapping datasets, which are created by randomly sampling from the training set with replacement (Supplementary Figure 1). Denote the parameters of $M$ trained models as $\{\theta^{\left( 1 \right)},\cdots,\theta^{(M)}\}$, $p(\theta)$ can be represented as the empirical distribution

$$p\left( \theta\right)=\frac{1}{M}\sum_{m=1}^{M} \delta(\theta-\theta^{(m)})$$

$p(z|x)$, the distribution of processed input given the raw input, can be simulated by applying multiple imputation. Denote the $S$ imputed inputs are represented as $\{z^{\left( 1 \right)},\cdots,z^{(S)}\}$.

However, this involves running $M$ trained models at the test time, which could be computationally expensive at the test time if $M$ is large. Therefore, we proposed to train two distillation models to approximate the estimated mean and variance across multiple models as follows

$$\mu_{distill}(z^{(s)})\approx\frac{1}{M}\sum_{m=1}^{M} f_{\theta^{\left( m \right)}}\left( z^{(s)} \right)$$

$$\sigma_{distill}^{2}(z^{(s)})\approx\frac{1}{\left( M-1 \right)}\sum_{m=1}^{M} {(f_{\theta^{\left( m \right)}}\left( z^{(s)} \right)-\hat{\mu}(z^{(s)}))}^{2}$$

where $\hat{\mu}\left( z^{(s)} \right)=\frac{1}{M}\sum_{m=1}^{M} f_{\theta^{\left( m \right)}}(z^{(s)})$

The model uncertainty can be estimated as

$$\hat{\sigma}_{model}^{2}\approx\frac{1}{S}\sum_{s=1}^{S} \sigma_{distill}^{2}(z^{(s)})$$

The prediction mean can be estimate as

$$\hat{\mu}\left( x \right)=\frac{1}{S}\sum_{s=1}^{S} \mu_{distill}(z^{(s)})$$

The 95% confidence interval based on the input uncertainty can be derived from the estimated $\hat{\mu}(x)$ and $\hat{\theta}_{model}^{2}$

$$\mathrm{CI}_{95\%}\left( r\left( x \right) \right)\approx[\hat{\mu}\left( x \right)-2\hat{\sigma}_{model},\hat{\mu}\left( x \right)+2\hat{\sigma}_{model}]$$

***Feature Impact Score Based on Input Uncertainty***

The input uncertainty can be estimated as follows

$$\hat{\sigma}_{input}^{2}\approx\frac{1}{S-1}\sum_{s=1}^{S} {(\mu_{distill}\left( z^{\left( s \right)} \right)-\hat{\mu}\left( x \right))}^{2}$$

We compute the feature impact score of the $d$-th feature $F_{d}$ as the reduction of the prediction variance induced by the input uncertainty when that feature’s value is fixed as the population mean across multiple imputations. To normalize the feature impact score, we consider the reduction of the prediction interval width, where the prediction interval is defined by the prediction mean and the input uncertainty.

$$\mathrm{FIS}\left( F_{d} \right)=w-w_{-d}$$

$$w=\mathrm{sigmoid}\left( \hat{\mu}+2\hat{\sigma}_{input} \right)-\mathrm{sigmoid}\left( \hat{\mu}-2\hat{\sigma}_{input} \right)$$

$$w_{-d}=\mathrm{sigmoid}\left( \hat{\mu}+2\hat{\sigma}_{input | z^{\left( s \right)}=const} \right)-\mathrm{sigmoid}\left( \hat{\mu}-2\hat{\sigma}_{input | z^{\left( s \right)}=const} \right)$$

Since feature impact score (FIS) would be high for missing features that 1) are predictive of the outcome; and 2) cannot be estimated accurately from measured features. We hypothesize after actively measuring the values of (missing) features having high FIS values, the classifier is more likely to make correct prediction for the patient. Therefore, FIS can be interpreted as patient-level feature importance score for missing values.

To validate this hypothesis, we run an experiment described below.

- For each test patient, remove 50% measured variables, the resulting data matrix is denoted as RemovedHalf. Denote the set of removed variables as R, which is constrained to exclude Heart Rate and three ventilation-related variables: FiO2, Mean Airway Pressure, Peak Inspiratory Pressure.
- For each test patient, compute FIS. for each feature in R. Recover the measured values of features with FIS values greater than a given threshold T, where the resulting data matrix is denoted as FIS.
- Recover the measured values of the same number of features randomly selected from R, the resulting data matrix is denoted as Random.
- The data matrix of the original test set is denoted as Original.

| Dataset | AUC (full test set) | AUC (20% test set) |
| --- | --- | --- |
| RemoveHalf | 0.786 | 0.745 |
| Random | 0.790 | 0.754 |
| FIS | 0.798 | 0.785 |
| Original | 0.841 | 0.831 |

Table 2: The AUC values of different input datasets when T=0.1.

Figure 4: Setting the threshold T=0.1, the top figure shows the probability of recovering a feature's value using FIS given its value was randomly removed. The bottom figure shows the results of the Random strategy.

The threshold T is set to be 0.1, which achieves a reasonable trade-off between the cost of measuring new features and the improvement of the classification performance. When setting T=0.1, among 8657 test samples, 20% samples were notified to measure new measures. Among these 20% test samples, each sample was notified to measure 1.2 features on average. The AUC values are shown in Supplementary Table 2. For each feature, we also compute the probability of recovering its value given its value was removed. The results of FIS and Random are shown in Supplementary Figure 4.

We can make the following observations

- FIS outperforms Random in terms both precision-recall curve and AUC values. This is because the variables identified by random sampling are not necessarily predictive of the outcome. In contrast, FIS can identify variables that are missing and predictive.
- Random would recover feature values with almost equal probability. In contrast, FIS would prefer recover feature values of Systolic BP, hemoglobin, temperature, Diastolic BP and Sodium.

Therefore, FIS can be interpreted as patient-level feature importance score for missing features.

***Model performance across hospitals***

We also evaluated the model performance across 27 different hospitals in our held-out test dataset. The model performs consistently well on most hospitals. Some key observations are 1) the breakeven PPV increases if the prevalence of unstable patients becomes higher in the hospital; 2) even after adjusting for prevalence (15%), there still exists variation of the model performance across multiple hospitals; 3) among 27 hospitals, the breakeven PPV of 22 hospitals is greater than 0.5. Although the model can be further adapted using data collected from each hospital, our results suggest that HSI can be deployed to a new hospital setting and expect to achieve reasonably good performance (with breakeven PPV greater than 0.5) with high probability (81%).

***Plausibility filters***

Vital signs including heart rate, blood pressures, and shock index were forward filled up-to 2 hours and laboratory measurements and ventilation settings were forward filled up to 26 hours (Supplementary Table 3). Some variables were capped to physiologically plausible ranges as shown in Supplementary Table 3, or otherwise treated as outliers and set to missing values if they were outside the plausible range.

| **Plausibility filters** | **Units** | **Expiration time** | **Low** | **High** | **Outliers** |
| --- | --- | --- | --- | --- | --- |
| Age | years | None | 17 | 90 | drop |
| CVP | mmHg | 26 | 0 | 50 | cap |
| Heart Rate | bpm | 2 | 0 | 200 | cap |
| Systemic Systolic BP | mmHg | 2 | 0 | 250 | drop |
| Non-Invasive Mean BP | mmHg | 2 | 0 | 250 | drop |
| Non-Invasive Systolic BP | mmHg | 2 | 0 | 250 | drop |
| AST | Units/L | 26 | 0 | 400 | cap |
| Bands | % | 26 | 0 | 100 | cap |
| Basophils | % | 26 | 0 | 20 | cap |
| Blood Urea Nitrogen | mg/dL | 26 | 0 | 500 | cap |
| Calcium | mg/dL | 26 | 0 | 20 | cap |
| CO2 | mmol/L | 26 | 0 | 50 | cap |
| Creatinine | mg/dL | 26 | 0 | 10 | cap |
| Eosinophils | % | 26 | 0 | 10 | cap |
| FiO2 | %, as fraction in [0,1] | 26 | 0 | 1 | cap |
| Glucose | mg/dL | 26 | 0 | 1000 | cap |
| Hematocrit | % | 26 | 10 | 70 | cap |
| Hemoglobin | g/dL | 26 | 2 | 22 | cap |
| Ionized Calcium | mg/dL | 26 | 0 | 20 | cap |
| Lactate | mg/dL | 26 | 0 | 100 | cap |
| Magnesium | mg/dL | 26 | 0 | 10 | cap |
| Mean Airway Pressure | mmHg | 26 | 0 | 50 | cap |
| PaCO2 | mmHg | 26 | 0 | 200 | cap |
| Peak Insp Pressure | mmHg | 26 | 0 | 100 | cap |
| Potassium | mmol/L | 26 | 2 | 10 | cap |
| PTT | seconds | 26 | 0 | 250 | cap |
| SaO2 | % | 26 | 0 | 100 | cap |
| Sodium | mmol/L | 26 | 100 | 200 | cap |
| Temperature | degrees (F) | 26 | 86 |  |  |
| Total Bilirubin | mg/dL | 26 | 0 | 50 | cap |
| WBC | k/uL | 26 | 0 | 60 | cap |
| Non-Invasive Shock Index | bpm/mmHg | 2 | 0 | 5 | cap |

Table 3: Features used in the HSI model and plausibility filters.

***Feature cross-correlation***

Figure 3: The Pearson correlation coefficient of all covariates from our train dataset. There is a high correlation between hematocrit and hemoglobin as both measures are dependent on plasma volume. We also observe a high correlation between blood pressures and between shock index and features used to compute shock index. The other covariates exhibit little to no correlation.

***Fluid and PRBC therapy triggers - Annotated***

| A segment was labeled “intervention” under any of the following conditions |
| --- |
| Administration of any quantity of any of the following inotropic and vasopressor medications:   - Dobutamine - Dopamine - Epinephrine - Norepinephrine - Phenylephrine - Vasopressin   Administration of Fluid Therapy (colloid or crystalloid) in the following dosages: [All indicate on-going fluid loss, volume instability or hemodynamically significant fluid deficit.]   - 2400 cc in eight hours. [This is 4 boluses of 10cc/kg or 2 (more significant boluses) of 20cc/kg for a 70kg adult] - 3000 cc in 12 hours [This is based on more practical terms (6 x 500 cc bolus) in 12 hrs or 1 500cc bolus every two hours. (bags of solution are either in 500cc or 1 liter volumes so the 3 liters equates to a number of bags of solution.] - 700 cc in one hour [This bolus represents more than the initial bolus recommended to test fluid responsiveness and indicates continuing need for extra fluid due to hypovolemia] - 1500 cc total in four hours [more than 20 cc/kg standard bolus in a short period of time represents typically administering 3 500 cc boluses in a relatively short period of time indicating on-going fluid loss or volume instability] - 500 cc twice in four hours [2x 500 cc bolus in a short time]   Administration of Packed Red Blood Cells (PRBCs) in either of the following dosages:   1. 250cc is the usual minimum volume of PRBC’s distributed by US blood banks for adult use. 2. 250cc’s of PRBC’s contains is equivalent to 2-3x this volume of whole blood 3. Blood loss replaced by the minimum dose (250cc’s) often represents known sources of loss such as documented surgical loss. 4. 500cc of whole blood loss is considered significant in the perioperative period 5. Transfusing 500 cc’s of PRBC’s is equivalent to replacing 1000 cc’s of whole blood loss.  - 800 cc PRBC over course of 24 hours [Infusing 800 cc’s of PRBC’s represents more than 3x the starting dose of PRBC replacement and indicates either on-going blood loss or a condition of extremely significant blood loss] - 500 cc in two hours followed by fluid therapy within 12 hours. (What qualifies as “fluid therapy” is described in this table, titled “Administration of Fluid Therapy.”) [Infusing 500 cc of PRBC’s equal to two times the minimum dose of PRBC’s and represents equivalent replacement of 1000 cc’s of whole blood. This is 2x what is considered significant perioperative blood loss. Continued need for additional fluid therapy for volume resuscitation beyond the significant (500cc) PRBC transfusion indicates likely on-going volume loss that is significant and is likely related to uncontrolled blood loss] - 500 cc PRBC not followed by fluid therapy within the following 24 hours. (What qualifies as “fluid therapy” is described in this table entry titled “Administration of Fluid Therapy.”) [Infusing 500 cc of PRBC’s equal to two times the minimum dose of PRBC’s and represents equivalent replacement of 1000 cc’s of whole blood.  This is 2x what is considered significant perioperative blood loss.] |

Table 4: Annotated reasoning for fluid therapy triggers.

Figure 4: Time between measurements of vital signs, laboratory measurements and ventilation settings in the eRI database.

***Missingness patterns***

We chose not to use missingness patterns for prognostic information because the pattern of measurements contains clinical concern. For example, clinical suspicion of instability can trigger new lab orders and simply the presence of certain test orders can trigger a change in the risk score that does not reflect a meaningful change in physiology. This is especially problematic when invasive measurements like CVP or invasive blood pressure measured through an arterial line is used as model inputs. Having an A-line can increase the risk of instability even if the blood pressure is stable. Another problem with learning from missingness patterns is the pattern of measurements can change over time or between institutions. For example, as measurements of fast troponin or bedside lactate increases in the future (or between institutions), the model would experience covariate shift in the measurement pattern. Encoding measurement patterns also reflects what the clinician already knows, and the model does not add new information about the patient’s physiology. Beulieu-Jone et. al (2021) provides a good justification: " if model performance is driven by the actions of the clinician (e.g., a test order) and not the underlying physiology (e.g., a test result) the model has the potential to confuse a clinician. For example, if a clinician orders a test because they suspect a condition with poor prognosis, but that test comes back normal, the clinician may rule out the condition, but the model may state the patient is at high risk based on the clinician’s original test order. ". For the above reasons, we attempted to remove sources of clinical concern to focus on the measurement values and not the measurement patterns.
